# Development and analytical validation of real-time PCR for the detection of *Streptococcus agalactiae* in pregnant women

**DOI:** 10.1186/s12884-020-03038-z

**Published:** 2020-06-09

**Authors:** Daniel F. Escobar, Diego A. Diaz-Dinamarca, Carlos F. Hernández, Daniel A. Soto, Ricardo A. Manzo, Pedro I. Alarcón, Camila H. Pinto, Diego N. Bastias, Carolayn N. Oberg-Bravo, Robert Rojas, Sebastián E. Illanes, Alexis M. Kalergis, Abel E. Vasquez

**Affiliations:** 1 Sección de Biotecnología, Instituto de Salud Pública de Chile, Santiago, Chile; 2 Millennium Institute on Immunology and Immunotherapy, Departamento de Genética Molecular y Microbiología, Facultad de Ciencias Biológicas, Pontificia Universidad Católica de Chile, Santiago, Chile; 3 Departamento de Bioquímica y Biología Molecular, Facultad de Ciencias Químicas y Farmacéuticas, Universidad de Chile, Santos Dumont 964, Independencia, 8380494 Santiago, Chile; 4 Sección Bacteriología del Departamento Biomédico, Instituto de Salud Pública de Chile, Santiago, Chile; 5 Escuela de Biotecnología y Escuela de Tecnología Médica, Facultad de Ciencias, Universidad Santo Tomas, Santiago, Chile; 6 Centro de Genómica y Bioinformática, Universidad Mayor, Santiago, Chile; 7 Department of Obstetrics and Gynecology, Faculty of Medicine, Universidad de los Andes, Santiago, Chile; 8 Department of Obstetrics and Gynecology, Clínica Dávila, Santiago, Chile; 9 Departamento de Genética Molecular y Microbiología, Facultad de Ciencias Biológicas, Departamento de Endocrinología, Facultad de Medicina, Pontificia Universidad Católica de Chile, Santiago, Chile; 10 Facultad de Medicina y Ciencia, Universidad San Sebastián, Providencia, Santiago, Chile; 11 Present address. Instituto de Salud Pública de Chile, Av. Marathon, Ñuñoa, 1000 Santiago, Chile

**Keywords:** Group B Streptococcus, qPCR, Surface immunogenic protein, Analytical validation, Bacterial detection

## Abstract

**Background:**

Group B *Streptococcus* (GBS) is the leading cause of invasive neonatal infection. In this study, we aimed to evaluate the analytical validation of qualitative real-time polymerase chain reaction (qPCR) as a means to detect GBS.

**Methods:**

Genomic DNA (gDNA) was purified from 12 ATCC bacterial strains, two belonging to GBS and the remainder acting as negative controls. Additionally, gDNA was isolated from 21 strains of GBS from various serotypes (Ia, Ib and II-VIII). All gDNA was used to evaluate the analytical validation of the qPCR method employing a specific Taqman probe. Inclusivity, exclusivity, anticipated reportable range, the limit of detection and robustness were evaluated. The methods used are described in international guidelines and other existing reports. The performance of this qPCR method for detecting GBS was compared to other microbiological methods used with vaginal-rectal samples from pregnant women.

**Results:**

Our qPCR method for detecting GBS was analytically validated. It has a limit of detection of 0.7 GE/μL and 100% analytical specificity. It detects all strains of GBS with the same level of performance as microbiological methods.

**Conclusion:**

Data suggest that this qPCR method performs adequately as a means to detect GBS in vaginal-rectal swabs from pregnant women.

## Background

*Streptococcus agalactiae,* also known as Group B *Streptococcus* (GBS), is a leading cause of sepsis and meningitis in newborns and young infants [1–3]. Colonisation in pregnant women is estimated to be 11 to 35%, and socioeconomic factors directly impact the percentage of carriers in the population [4]. Even though screening programs and intrapartum antibiotic prophylaxis has reduced early neonatal onset of disease, this bacterium remains the leading cause of infection in newborns in developing countries [2, 5, 6].

The Centers for Disease Control and Prevention (CDC) recommends that vaginal and rectal swabs be used to inoculate a selective enrichment medium such as Lim broth and subsequently subculture the product on blood agar plates [2]. For better sensitivity or test efficiency, an alternative such as Granada medium could be used. In this case, a color change indicates GBS growth [7]. Unfortunately, this approach has variable sensitivity [8–10] and is associated with long turn-around times (18–72 h). A more rapid test that retains high sensitivity and specificity compared to conventional culture-based methods is real-time polymerase chain reaction (qPCR) [11]. This method will not fail to detect non-hemolytic GBS, as do culture-based methods. Non-hemolytic and non-pigmented GBS are reportedly found in 1 to 4% of pregnant women [12].

It is crucial that sensitive and accurate qPCR strategies be developed so that the efficacy of intrapartum antibiotic prophylaxis can be determined [7]. Also essential is the analytical validation of the method by evaluating parameters such as analytical specificity (inclusivity and exclusivity), analytical sensibility (anticipated reportable range and limit of detection), linearity, efficiency and robustness, when used for detection. Quantitative methodologies require estimates of precision, accuracy and limits of quantification [13]. A validated analytical method confirms that GBS detection will be an accurate, precise and reliable measure of potency.

A surface immunogenic protein (SIP) is reportedly exposed at the surface of intact GBS cells no matter the serotype [14, 15]. Because the *sip* gene is conserved in GBS, qPCR strategies have been used to successfully detect GBS in clinical studies [16–20]. However, an analytically validated qPCR assay for GBS using the target *sip* gene has not yet been reported.

We developed a qPCR method that uses the *sip* gene as its target (Intellectual Property PCT/IB2017/056506), and herein, we report the analytical validation and standardisation of that method using Clinical and Laboratory Standards Institute (CLSI) guidelines MM17-A (Validation and Verification of Multiplex Nucleic Acid Assays) and EP17-A (Protocols for Determination of Limits of Detection and Limits of Quantitation and Evaluation) [21–23]. Based on the work of others, the relevant parameters were identified as: selectivity (inclusivity and exclusivity), anticipated reportable range, the limit of detection (LOD), linearity, efficiency and robustness [24–28].

## Methods

### Design of oligonucleotides

Oligonucleotides were designed using free online software (Amplifx 1.7.0). They were based on the GBS *sip* gene, which codes for the GBS SIP (GenBank accession no.KX363665.1). After in silico analysis using Blast, the set of oligonucleotides showing the greatest sensitivity and specificity were: forward (SIP-F), GTTCCAGCAGCTAAAGAGGAAG; reverse (SIP-R), CCGGTGCTACTTTAGCTACTGG; and probe (SIP-P), FAM-CACCAGCTTCTGTTGCCGCTGAAACACCAGC-BHQ1 (Supplementary Fig. 1). A PCR product of 118 bp was obtained (Supplementary Fig. 2) (Intellectual Property PCT/IB2017/056506).

### Collection of clinical samples

Vaginal-rectal swabs were collected from pregnant women at a private health center and sent to the Health Public Institute of Chile ISPCH for GBS detection. All samples (*n* = 103) were collected between the 35th and 37th weeks of pregnancy. The study protocol was approved by the Ethics Committee of Clínica Dávila, and written informed consent was obtained from all study participants, agreeing that this work can be published. Samples were collected and submitted to Stuart transport medium at room temperature at the health center. After 2 hours, samples were cooled to 4 °C with ice packs, collected in a tertiary container and sent for analysis.

### Collection strains

To perform standardisation and to determine the analytical parameters of the methodology, specific reference strains were obtained from the American Type Culture Collection (ATCC): *Streptococcus agalactiae* ATCC 12403, *Streptococcus agalactiae* ATCC 13813, *Streptococcus pyogenes* ATCC 21547, *Staphylococcus aureus* ATCC 25923, *Enterococcus faecalis* ATCC 29212, *Streptococcus pneumoniae* ATCC 49619, *Escherichia coli* ATCC 25922, *Klebsiella pneumoniae* ATCC 700603 and *Klebsiella aerogenes* ATCC 13048. Three non-ATCC strains, confirmed by the ISPCH, were included: *Staphylococcus saprophyticus, Proteus* spp. and *Serratia marcescens*. Isolates of axenic cultures of nine serotypes of GBS (*n* = 21) from patients with invasive infections (received from 2013 to 2018 by the Bacteriology Reference Laboratory of the ISPCH for confirmation) were also used.

### Pretreatment of vaginal-rectal swabs

**Fig. 1:**
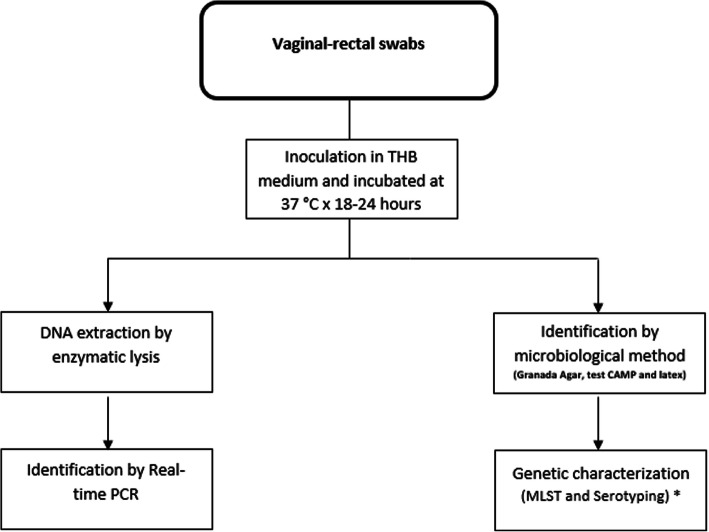
Working scheme of the clinical vaginal-rectal swab specimens collected (*n* = 103). *Genetic characterisation data not shown

All vaginal swabs were inoculated in 3 mL selective Todd-Hewitt broth and supplemented with nalidixic acid (15 μg/mL; Sigma-Aldrich, St. Louis, MO, USA) and gentamicin (8 μg/mL; Sigma-Aldrich), then were incubated for 18 to 24 h at 37 °C in an atmosphere of 5% CO_2_. This procedure was duplicated; one was analysed using qPCR and the other using a microbiological method (Granada agar, Christie, Atkins and Munch-Peterson [CAMP] test and latex agglutination; Fig. 1).

### DNA extraction from clinical samples

Bacterial cultures were centrifuged at 10,000 g for 10 min. Then, from each pellet, a portion with a sterile tip was resuspended in 200 μL 5% Chelex-100 (Sigma-Aldrich), 1 μL commercial numan DNA (10 ng/μL) and 10 μL lysozyme (5 mg/mL, Biological, Salem, MA, USA). The mixture was incubated at 37 °C for 15 min, then, 10 μL proteinase K (2 mg/mL, Merck) was added. Incubation at 56 °C for 15 min was followed by a one-step inactivation (95 °C for 15 min). Finally, the lysate bacterial culture was centrifuged at 10,000 *g* for 3 min then stored at − 20 °C until qPCR analysis.

### DNA isolation from each strain

All strains used for the determination of analytical parameters (previously characterised strains, both ATCC and non-ATCC) were cultured in blood agar, and gDNA was then extracted using E.Z.N.A. A manual bacterial DNA kit (OMEGA Bio-Tek, Norcross, GA, USA) was applied following manufacturer instructions. Genetic material was eluted using 100 μL eluent, then samples were screened for GBS using qPCR. The gDNA was quantified using a DS-11 spectrophotometer (Denovix, Wilmington, DE, USA). To determine the number of genomic equivalents, one GBS genome (2.16 Mbp) was obtained from the NCBI Reference Sequence (GenBank accession no. NC_004116.1), and its mass was calculated using: bp*M*N_A_, where bp is the size of the genome in bp, M is its molar mass in g/mol and N_A_ is Avogadro’s number in mol^− 1^. One genomic equivalent of GBS was found to have a mass of 2.4 fg.

### Real-time PCR procedure

Assays were performed using 1 μL gDNA template and a Stratagene Mx3000P thermocycler (Agilent Technologies, Santa Clara, CA, USA). To detect GBS, 1 μL of each primer (4 μM stock), 1 μL probe (2 μM stock) and 10 μL Brilliant Multiplex Real-Time PCR Master Mix (Agilent Technologies, Santa Clara, CA, USA) were used. The optimal reaction conditions were found to be 95 °C for 10 min followed by 40 cycles at 95 °C for 15 s, then 60 °C for 1 min. A cycle threshold (*Ct*) value of 35 was used as the cut-off for positive fluorescence detection of the target.

### Analytical specificity

Analytical specificity was evaluated in terms of selectivity, given the responsiveness of the method to selectively identify DNA sources using the *sip* target (GBS DNA) in non-GBS sources. This feature included:

#### Inclusivity

Purified gDNA from *S. agalactiae* ATCC 12403 was prepared at concentrations of 24, 120 and 240 fg/μL (10, 50 and 100 GE/μL) for nine available serotypes (Ia, Ib, II, III, IV, V, VI, VII and VIII). Each was run in duplicate.

#### Exclusivity

Purified DNA from *S pyogenes*, *S. aureus*, *E. faecalis*, *S. pneumoniae*, *E. coli*, *K. pneumoniae*, *K. aerogenes, S. saprophyticus, Proteus* spp. and *S. marcescens* was prepared at concentrations of 1, 10, 100 and 1000 pg/μL for each. Each was run in duplicate.

### Analytical sensitivity

Analytical sensitivity, the lowest amount of analyte that can be detected with (a stated) probability was evaluated. However, exact values were not the focus. This feature includes:

#### Anticipated reportable range

Serial dilutions of gDNA from *S. agalactiae* ATCC 12403 resulted in final concentrations between 4.8E04 and 0.48 fg/μL (20,000 to 0.2 GE/μL). Each dilution was amplified six-fold in four qPCR assays, providing 24 data points for the standard curve. Assigned (theoretical) versus measured values were converted to log_10_ (GE/μL) and were then plotted for linear regression analysis.

### Limit of detection

According to the CLSI guidelines, the LOD is the lowest DNA concentration that provides at least 95% detectable results. Four concentrations of purified gDNA were adjusted to 9.6, 19.2, 38.4 and 76.8 fg/μL (32 to 4 GE/μL) and were analysed using qPCR. A blank (pooled negative samples) was also included. A total of 60 points and blanks were obtained over the course of five consecutive days. According to the CLSI protocol, to determine LOD, the limit of blank (LOB), the highest qPCR measures expected in samples devoid of analyte, must be determined.

### Robustness

To evaluate a test’s robustness, combinations between factors in standardised and alternative conditions were generated. The factors analysed were: master mix, concentration of primers, concentration of probe, upper alignment temperature, lower alignment temperature, equipment and operator. (Table 1).

**Table 1:** Robustness test as described by Broeders et al. (2014)

Combination No								
Factor value	1	2	3	4	5	6	7	8
**A or a**	A	A	A	A	a	a	A	a
**B or b**	B	B	b	b	B	B	B	b
**C or c**	C	c	C	c	C	c	C	c
**D or d**	D	D	d	d	d	d	D	D
**E or e**	E	e	E	e	e	E	E	E
**F or f**	F	f	f	F	F	f	f	F
**G or g**	G	g	g	G	g	G	G	g

(A) Standardised master mix; (a) alternative master mix; (B) standardised concentration of primers; (b) lower concentrations of primers; (C) standardised concentration of probe; (c) lower concentration of probe; (D) standardised alignment temperature; (d) upper alignment temperature; (E) standardised alignment temperature; (e) lower alignment temperature; (F) equipment used for validation; (f) alternative equipment; (G) operator 1; (g) operator 2

### Precision

Three concentrations of purified gDNA from *S. agalactiae* ATCC 12403 were adjusted to 4.8, 9.6 and 240 fg/μl (2, 4 and 100 GE/μL or 0.3, 0.6 and 2 log_10_ GE/μL). Precision was found using CLSI recommendations (the 20x2x2 method, performing 2 daily runs of duplicates on 20 different days). The estimates of within-laboratory precision standard deviation (*S*_*WL*_*)* and repeatability standard deviation (*S*_*R*_) were calculated using: *S*_*WL*_ = [Ѵ_day_ + Ѵ_run_ + Ѵ_error_]^1/2^; *S*_*R*_ = [Ѵ_error_]^1/2^, respectively, where Ѵ_day_, Ѵ_run_ and Ѵ_error_ correspond to variance components of the day, run, and error, respectively. The variance component was calculated using MS, the mean of the square: Ѵ_error_ = MS_error_; Ѵ_run_ = MS_run_ – MS_error/_n_rep_ (number of replicates per run); Ѵ_day_ = MS_day_ – MS_run_/n_run_n_rep_ (number of runs per day; number of replicates per run). The sum of the squares, provided by the two-way nested analysis of variance, was used to calculate MS, and DF, the degrees of freedom (for each source of variation), was obtained as: DF_day_ = n – 1; DF_run_ = (n_run_ – 1)n_day_; DF = N – n_daynrun_; and DF_total_ = N − 1, where n_day_ = 20 days; n_run_ = 2 runs per day, n_rep_ = 2 replicates per run and *N* = 80 results per sample.

Two-sided 95% confidence intervals (CIs) were calculated using the standard deviations of the degrees of freedom associated with repeatability (*df*_*R*_) and the degrees of freedom associated with the laboratory (*df*_*WL*_), where α_day_ = 0.25, α_run_ = 0.25 and α_error_ = 0.5, using the following formulas, respectively: *df*_*R*_ = N - n_daynrun_ and *df*_*WL*_ = (α_day_ MS_day_ + α_run_ MS_run_ + α_error_ MS_error_)^2^ / (α_day_ MS_day_)^2^ / DF_day_ + (α_run_ MS_run_)^2^ / DF_run_ + (α_error_ MS_error_)^2^ / DF_error_. The CIs of repeatability were then computed using: *S*_*R*_ [*df*_*R*_ / χ21-α/2]^2^ and *S*_*R*_ [*df*_*R*_ / χ2α/2]^2^. The CIs of within-laboratory precision were computed using: *S*_*WL*_ [*df*_*WL*_ / χ21-α/2]^2^ and *S*_*WL*_ [*df*_*WL*_ / χ2α/2]^2^. Values obtained were re-expressed as %CV using the data (Supplementary Fig. 3).

### Quality assurance of the results

Parallel to the clinical samples, commercial exogenous internal controls were run to rule out errors in the DNA purification process: A TaqManRNase P Detection Reagent Kit (Applied Biosystems, CA, USA) was applied following manufacturer instructions. The thermal profile conditions were the same as those used for amplification of the *sip* gene.

### Statistical analysis

The GraphPad Prism 6 software (GraphPad Software, La Jolla, CA, USA) was used to carry out ANOVA analysis to determine the precision of the method and normal data distribution tests for choose the equation that determined the LOB and LOD, according to guidelines provided by CLSI documents EP05A3 and EP17-A, respectively.

## Results

### Determining the analytical parameters of the qPCR assay of GBS

**Fig. 2:**
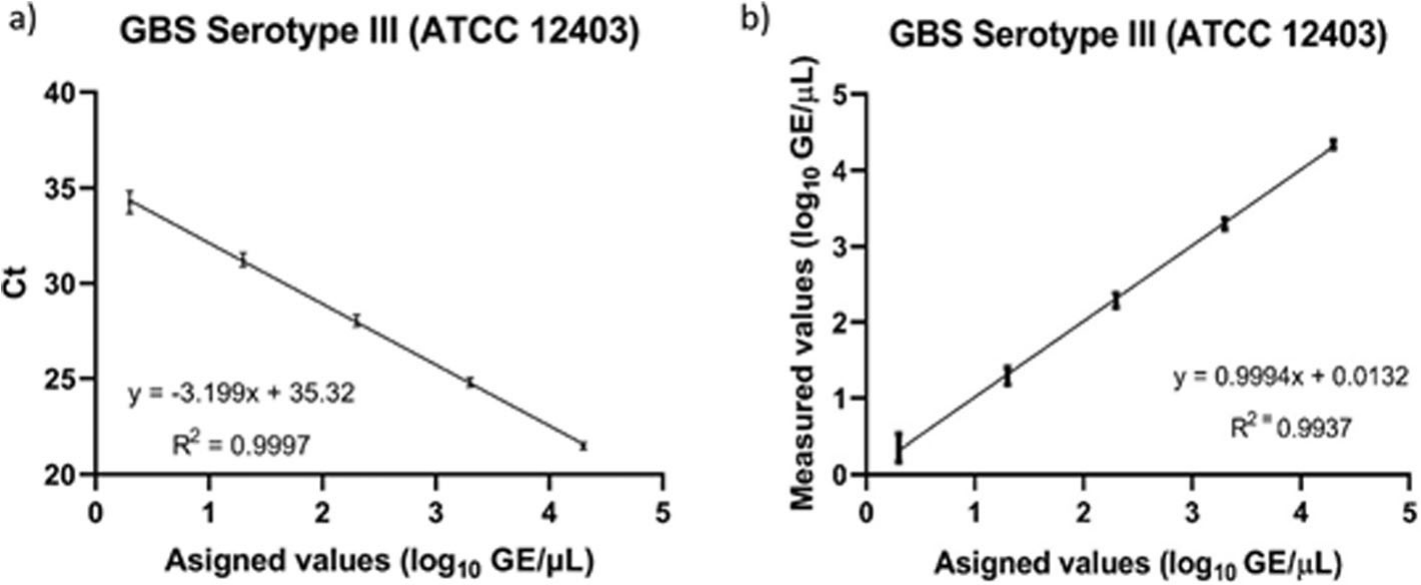
Determination of the anticipated reportable range. **a** Standard curve between the value assignment (log_10_ GE/μL) and cycle threshold, as obtained from the points used to calculate real-time polymerase chain reaction efficiency (*ε*). **b** Linear regression of the points on the standard curve between the assigned values and those obtained from measurements (log_10_ GE/μL). Also shown is the equation obtained from the model

Analytical validation of the qPCR assay was obtained using the parameters specified. The first to be determined was necessarily the anticipated reportable range. The high and low concentrations were measured in GEs per microliter. The anticipated reportable range, determined by constructing two standard curves, showed a linear correlation between the expected and obtained values (*R*^*2*^ = 0.9937) and between the assigned values for *Ct* and those found by the analytical software (*R*^*2*^ = 0.9997) when the analyte was between 20E04 and 2 GE/μL (Fig. 2). The sixth point on the curve (0.2 GE/μL) was not amplified in any of the 24 replicates run during the four tests designed to determine this parameter. The *Ct* values at the lowest detectable dilution (2 GE/μL) ranged from 33.06 to 35.36. The LOD (0.7 GE/μL) correlated with the results of the anticipated reportable range, which lacked a detection signal when dilution was between 2 and 0.2 GE/μL.

**Fig. 3:**
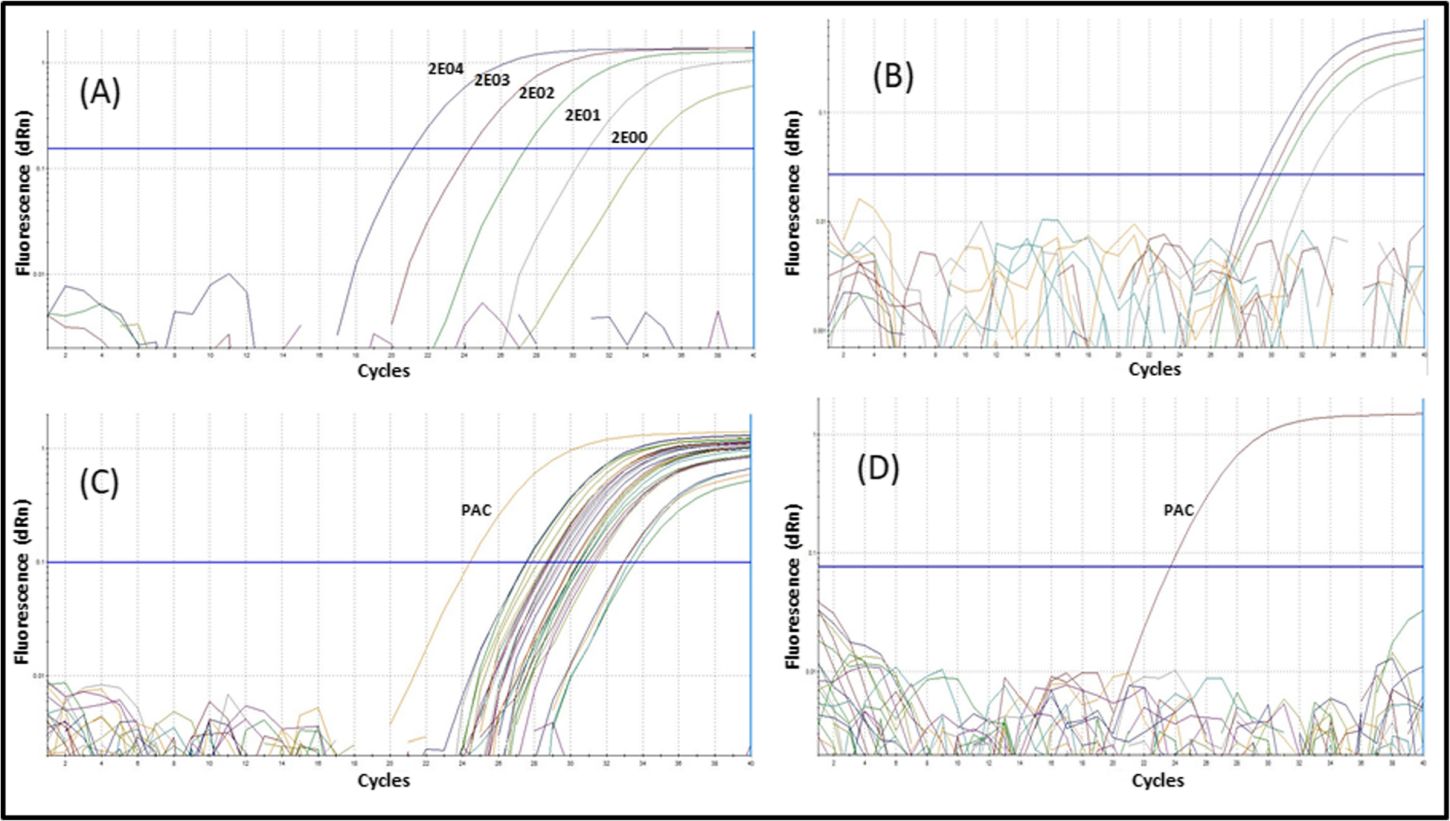
Amplification curves of measured parameters. Positive, negative and no-template controls were included in all reactions. **a** Standard curve (2E04–2 GE/μL) constructed to determine the anticipated reportable range. **b** Amplification of curves (32–4 GE/μL) used to determinate the limit of detection. The pool of samples lacking GBS strains did not produce an amplification signal. **c** Curves showing the amplification signals from all GBS serotypes tested, used to determine the inclusivity parameter. **d** Curves for negatives, samples from non-GBS strains, used to determinate the exclusivity parameter. *Note.* PAC: positive amplification control for GBS

Exclusivity tests demonstrated that qPCR did not detect DNA in the range of 1 to 1000 pg/μL for *S. pyogenes* ATCC 21547, *S. aureus* ATCC 25923, *E. faecalis* ATCC 29212, *S. pneumoniae* ATCC 49619, *E. coli* ATCC 25922, *K. pneumoniae* ATCC 700603, *K. aerogenes* ATCC 13048, *S. saprophyticus*, *Proteus* spp. or *S. marcescens*. The 21 strains that corresponded to 9 of 10 available serotypes (Ia, Ib and II to VIII) and the γ-hemolytic strain *S. agalactiae* ATCC 13813 (serotype II) were detected at low DNA concentrations (24 fg/μL or 10 GE/μL), revealing that qPCR is inclusive and able to independently detect the various strains of the serotype. Each parameter was found to have an analytical specificity of 100% (Table 2; Fig. 3). Robustness was shown: Every combination produced an amplification signal, and *Ct* values remained between 32 and 34.

**Table 2:** Summary of results for each parameter evaluated in the analytical validation

Validation parameters	***sip***-qPCR
**Inclusivity (detectable for qPCR) (fg/μL)**	
GBS serotype Ia (n = 3)	24
GBS serotype Ib (n = 2)	24
GBS serotype II (n = 4)	24
GBS serotype III (n = 2)	24
GBS serotype IV (n = 3)	24
GBS serotype V (n = 2)	24
GBS serotype VI (n = 2)	24
GBS serotype VII (n = 3)	24
GBS serotype VIII (n = 1)	24
**Exclusivity (non-detectable qPCR) (0.1 to 1000 pg/μL)**	
*S. pyogenes* ATCC 12384	ND
*S. aureus* ATCC 25923	ND
*E. faecalis* ATCC 29212	ND
*S. pneumoniae* ATCC 49619	ND
*E. coli* ATCC 25922	ND
*K. pneumoniae* ATCC 700603	ND
*K. aerogenes* ATCC 13048	ND
*S. saprophyticus*	ND
*Proteus* spp	ND
*S. marcescens*	ND
**Reportable range (GE/μL)**	
GBS ATCC 12403 serotype III	20.000-2
**Limit of detection (GE/μL)**	
GBS ATCC 12403 serotype III	0.7
**Robustness**	
GBS ATCC 12403 serotype III (% correct positives)	100
Samples non-GBS (% correct negatives)	100
**PCR efficiency (ε) (%)**	105.4
**Linearity (*R***^***2***^**)**	0.9937

*fg/μL* femtograms in 1 microliter, *pg/μL* picograms in 1 microliter, *GE/μL* genomic equivalents in 1 microliter, *ND* not detectable, *R*^*2*^ correlation coefficient

### Comparative analysis between the microbiological method and qPCR for detecting GBS in vaginal-rectal swabs from pregnant women

**Fig. 4:**
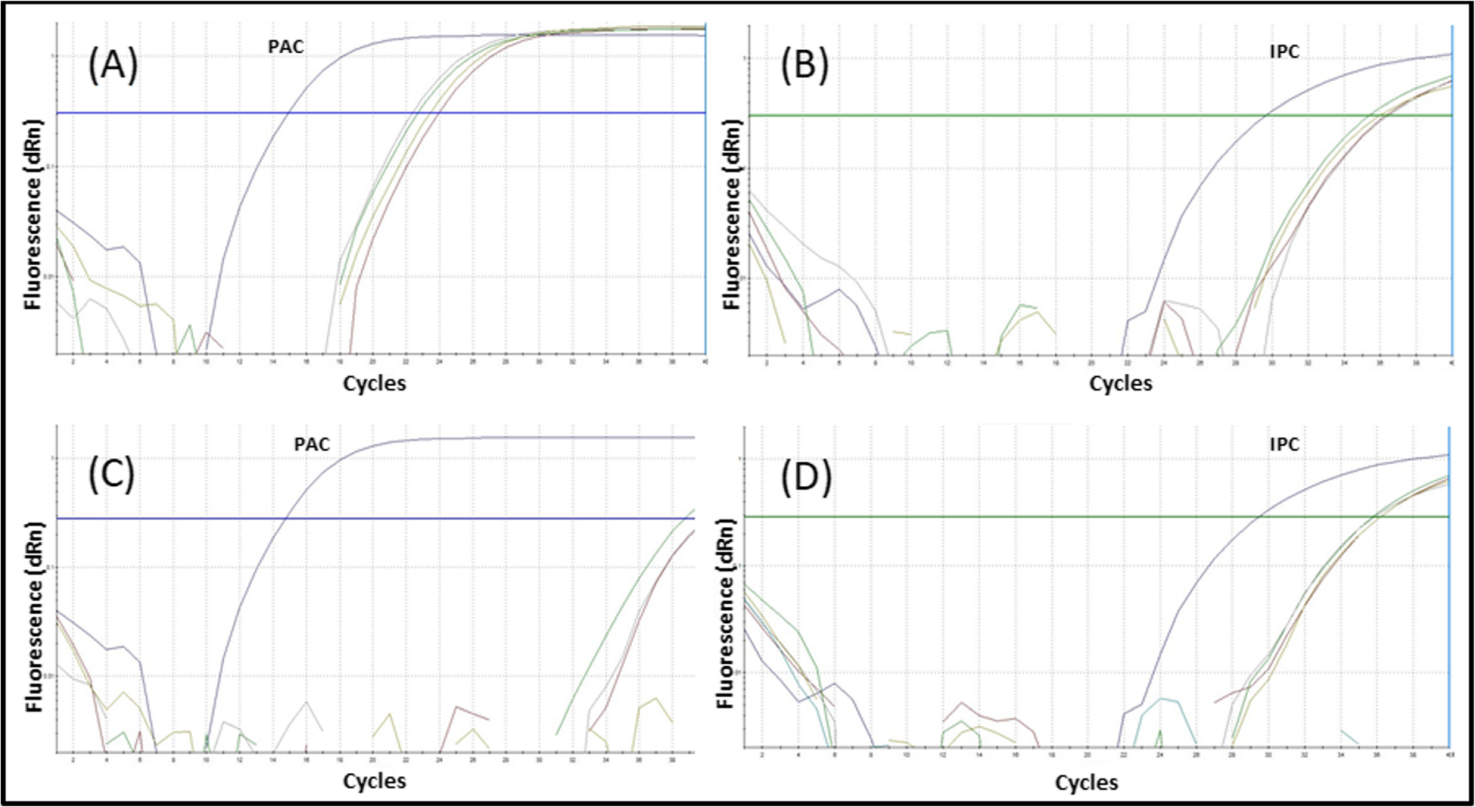
Amplification plots with representative curves for positive and negative samples. Positive, negative and no-template controls were included in all reactions. **a** Amplification curves for the *sip* gene fragment from positive samples. **b** Amplification curves for internal positive controls from positive samples. **c** Amplification curves for the *sip* gene fragment from negative samples. **d** Amplification curves for internal positive controls from negative samples *Note.* IPC: internal positive control (exogenous, gene coding for the RNA subunit of ribonuclease P); PAC: positive amplification control for GBS

In total, 103 vaginal-rectal swabs (clinical samples) were analysed to confirm the presence of GBS. Samples were analysed blindly, and the results of the qPCR assays were compared to those found using a microbiological method. With the latter (a Granada agar plate, CAMP test and latex agglutination), GBS was found in 15 samples. The same was true for qPCR (Table 3; Fig. 4).

**Table 3:** Confirmatory analysis of 103 clinical samples obtained from pregnant women

*Result*	*Tests*		*Total of samples analyzed*
	***qPCR***	***Microbiological Method***	
Positive	15	15	103
Negative	88	88

## Discussion

A qPCR method to directly identify GBS from vaginal-rectal swabs after an enrichment process in Todd-Hewitt broth was successfully developed. This methodology provides results that are the same as those obtained using the microbiological method routinely used in clinical laboratories. The qPCR methodology is quick and easy to interpret by technical health personnel, and it can be incorporated into a working algorithm to identify GBS. Analytical validation was performed by extracting gDNA using proteinase K, lysozymes and Chelex-100, reducing costs compared to a genomic DNA purification kit. This methodology is advantageous compared to others because it can detect low concentrations of genomic DNA (2 GE/μL) and can potentially be used as a GBS screening tool at delivery directly from a vaginal swab. A disadvantage is that the CDC does not recommend this practice. Moreover, molecular methods are limited in GBS detection because antibiotic resistance genes have not yet been incorporated into the PCR primer set. Doing so could enable the development of a rapid test that will provide additional information such as antibiotic susceptibility, for example, in the context of a penicillin allergy [29]. Meanwhile, microbiological analyses are recommended for determining susceptibility.

Several molecular diagnostic tools are emerging as potential candidates for more rapid identification of invasive GBS disease or to rapidly identify women colonised with GBS [29]. Rapid diagnostic tests for invasive diseases include the MinIon®, loop-mediated isothermal amplification [17] and optical immunoassays. Those for rapid intrapartum colonisation screening include PCR-based methods [30]. However, publicly available data for the analytical validation of these methods for GBS screening in a clinical setting are lacking. Our analytical validation method assessed the performance of qualitative qPCR strategies based on the TaqMan probe for GBS detection, and it was conducted according to international guidelines. The *sip* gene from GBS was used as a molecular target, and inclusivity and exclusivity were estimated using gDNA from stocks of various bacterial strains. The analytical value of this methodology is in its anticipated reportable range (20,000 to 2 GE/μL) and its LOD (0.7 GE/μL). Additionally, it has a high level of precision in repeatability conditions, reinforcing its applicability (Supplementary Fig. 3, Supplementary Table 1 and Supplementary Table 2). During pregnancy, recto-vaginal colonisation of GBS is not persistent; it is characterised by a high rate of new acquisitions and a loss of colonisation over the course of pregnancy [31]. A culture-based method applied to samples taken between the 35th and 37th weeks of gestation might fail to detect all colonisations at delivery because GBS could infect between sampling and the onset of labor. For this reason, an intrapartum screening strategy using a qPCR method to identify and quantify GBS would help avoid risk-factor-based screening [32]. Compared to GBS cultures in enriched media, PCR has better sensitivity and specificity, ranging from 62.5–100% and 84.6–100%, respectively [31].

To date, the gold standard for detecting GBS is the microbiological method, which is inexpensive and easy to perform. However, it fails to detect non-hemolytic GBS strains, and β-hemolysis could be difficult to observe [7]. Therefore, negative samples should be further tested for other GBS strains using either a subculture on Granada agar plates, direct latex agglutination testing or (increasingly) molecular methods such as qPCR. Several clinical studies have been described for GBS detection by qPCR targeting the sip gene and have reported sensitivity between 95.4–97% and specificity between 84.6–99% (Supplementary Table 3); high sensitivity and specificity previously describes may be explained by the fact that the sip gene is conserved among all serotypes. On the other hand, none have declared analytical validation of the qPCR methodology, which could considerably improve future clinical validation studies. Although positive and negative controls are important when validating a result, a greater understanding of the methodology used, gained through analytical validation, is imperative. Our analytical validation parameters describe a trend that is similar to those seen with pathogens such as *Trypanosoma cruzi* (LOD, 0.23 and 0.70 parasite equivalents/mL) [13], *Plasmodium knowlesi* (analytical sensitivity, 10 copies/μL) [33] and the American H7 virus (10E3 and 10E4 gene copies per reaction) [34]. Finally, the availability of this standardised and validated qPCR method provides new possibilities for screening women in labor and helps to improve the detection of GBS strains using microbiological methods.

Using qPCR to detect the *sip* gene has been reported elsewhere, and sensitivity and specificity were acceptable [16, 19, 20, 35, 36] (Supplementary Table 3). However, we established the limitations of this amplification process using analytical validation. Furthermore, our methodology, when compared with traditional methods currently considered to be the gold standard for GBS detection, shows 100% correlation. Nevertheless, the potential of this test should be explored because it could reduce swab incubation time or possibly allow direct extraction of genetic material. The technique developed here allows GBS to be detected in polymicrobial samples, and its specificity is superior compared to the microbiological method, which requires 3 to 5 days for incubation.

## Conclusion

This kit accurately detects the bacterial agent GBS, it avoids cross-reactions with related bacteria and it provides 100% analytical specificity. It has a low LOD (0.7 GE/μL or 14 genomes per reaction) and optimal performance, and it meets analytical needs, as shown in its linearity and efficiency data, which correlates with current screening methods. These preliminary results are favorable and provide the basis for a study with a greater sample size, which could confirm the effectiveness of this test using clinical validation.

## Supplementary information

**Additional file 1: Table S1.** The analysis of variance summary for data collected from three concentrations used to estimate the precision parameter. *Note.* DF: degrees of freedom; MS: mean of the squares; SS: sum of the squares.

**Additional file 2: Table S2.** Summary of the data from three concentrations used for estimating confidence intervals for repeatability and inter-laboratory precision. Abbreviation: CI: 95% Confidence interval. Repeatability results: at 2 GE/μL, 9.5% CV [CI, 7.8–12.2]; at 4 GE/μL, 5.9% CV [CI, 4.9–7.6]; and at 100 GE/μL, 1.8% CV [CI, 1.5–2.4]. Intra-laboratory precision results: at 2 GE/μL, 12% CV [CI, 9.9–14.1]; at 4 GE/μL, 8.8% CV [CI, 6.6–9.3]; and at 100 GE/μL, 2.3% CV [CI, 1.9–2.7].

**Additional file 3: Table S3.** Results of various studies of qPCR analysis using the *sip* gene as the target for GBS detection.

**Additional file 4: Figure S1.** Alignment of the *sip* gene and oligonucleotides (118 bp).

**Additional file 5: Figure S2.** Detection of the *Streptococcus agalactiae sip* gene using qPCR. **A** Typical amplification plot using 2300 GE/μL as the DNA template. dRn, fluorescent signal using ROX as a passive reference. **B** The polymerase chain reaction (PCR) product from **A** was fractionated on a 2% agarose gel and visualised using gel red staining. Lane 1, DNA molecular weight marker, through analytical validation bp; lanes 2 and 3, PCR product, 2300 GE/μL; lanes 4 and 5, no-template control.

**Additional file 6: Figure S3.** Daily runs used to estimate within-laboratory precision. Three bacterial concentrations from ATCC 12403 (2, 4 and 100 GE/μL), represented as log_10_ GE/mL, were evaluated using a single-site design of 20x2x2 according to the CLSI EP05-A3 guidelines. **A** Values (log_10_ GE/mL) for the 2 GE/mL concentration obtained from four daily replications over twenty days. **B** Values (log_10_ GE/mL) for the 4 GE/mL concentration obtained from four daily replications over twenty days. **C** Values (log_10_ GE/mL) for the 100 GE/mL concentration obtained from four daily replications over twenty days.

## Data Availability

The datasets used during the current study are available from the corresponding author on reasonable request, but most of the data generated during this study are included in this published article and its supplementary information files.

## Abbreviations

ATCC: American Type Culture Collection
bp: base pair
CAMP: Christie, Atkins and Munch-Peterson
CDC: Centers for Disease Control and Prevention
CLSI: Clinical and Laboratory Standards Institute
*Ct*: Cycle threshold
DNA: Deoxyribonucleic acid
GBS: Group B *Streptococcus*
LOD: Limit of detection
LOB: Limit of blank
Mbp: Megabase pair
PCR: Polymerase chain reaction
qPCR: Real-time PCR
SIP: Surface immunogenic protein
