# Supplementary figures and images for: Development and analytical validation of real-time PCR for the detection of Streptococcus agalactiae in pregnant women
Source: BMC Pregnancy Childbirth. 2020 Jun 9;20:352. doi: 10.1186/s12884-020-03038-z (PMC7285471; doi:10.1186/s12884-020-03038-z)

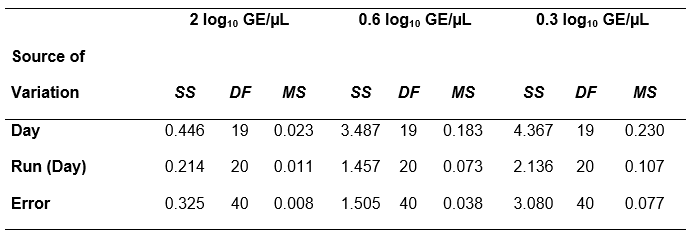

Supplement: Supplementary file 1 — Additional file 1: Table S1. The analysis of variance summary for data collected from three concentrations used to estimate the precision parameter. Note. DF: degrees of freedom; MS: mean of the squares; SS: sum of the squares. [file 12884_2020_3038_MOESM1_ESM.tif]

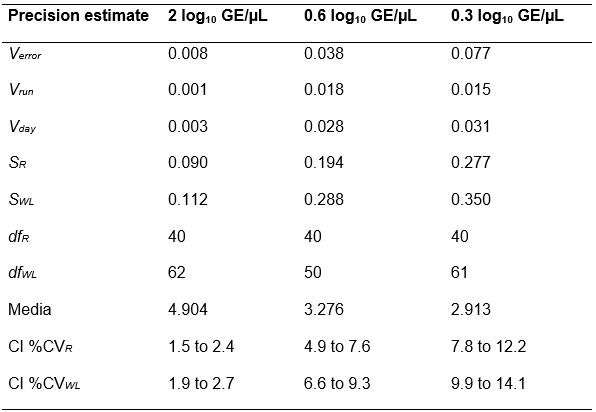

Supplement: Supplementary file 2 — Additional file 2: Table S2. Summary of the data from three concentrations used for estimating confidence intervals for repeatability and inter-laboratory precision. Abbreviation: CI: 95% Confidence interval. Repeatability results: at 2 GE/μL, 9.5% CV [CI, 7.8–12.2]; at 4 GE/μL, 5.9% CV [CI, 4.9–7.6]; and at 100 GE/μL, 1.8% CV [CI, 1.5–2.4]. Intra-laboratory precision results: at 2 GE/μL, 12% CV [CI, 9.9–14.1]; at 4 GE/μL, 8.8% CV [CI, 6.6–9.3]; and at 100 GE/μL, 2.3% CV [CI, 1.9–2.7]. [file 12884_2020_3038_MOESM2_ESM.tif]

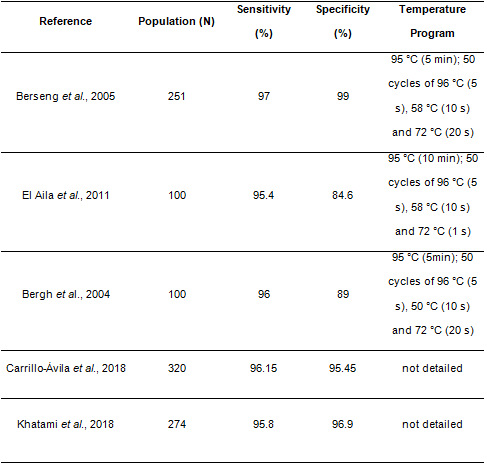

Supplement: Supplementary file 3 — Additional file 3: Table S3. Results of various studies of qPCR analysis using the sip gene as the target for GBS detection. [file 12884_2020_3038_MOESM3_ESM.tif]

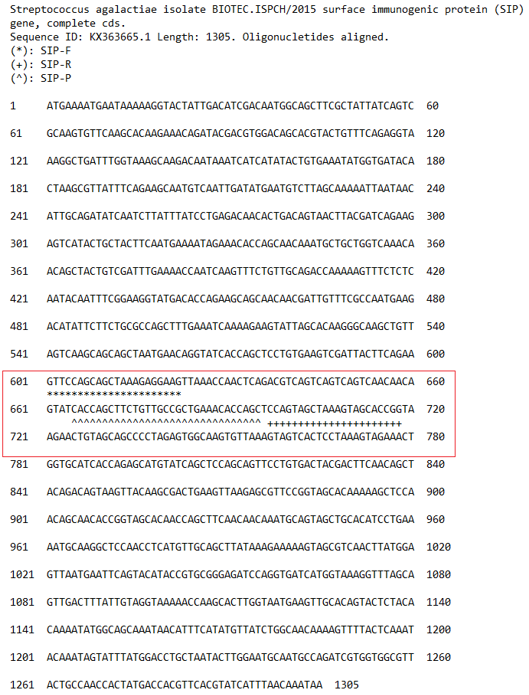

Supplement: Supplementary file 4 — Additional file 4: Figure S1. Alignment of the sip gene and oligonucleotides (118 bp). [file 12884_2020_3038_MOESM4_ESM.tif]

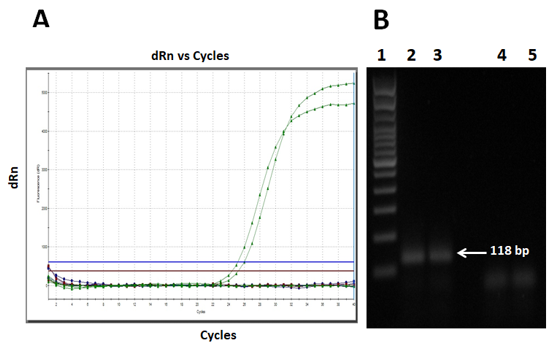

Supplement: Supplementary file 5 — Additional file 5: Figure S2. Detection of the Streptococcus agalactiae sip gene using qPCR. A Typical amplification plot using 2300 GE/μL as the DNA template. dRn, fluorescent signal using ROX as a passive reference. B The polymerase chain reaction (PCR) product from A was fractionated on a 2% agarose gel and visualised using gel red staining. Lane 1, DNA molecular weight marker, through analytical validation bp; lanes 2 and 3, PCR product, 2300 GE/μL; lanes 4 and 5, no-template control. [file 12884_2020_3038_MOESM5_ESM.tif]

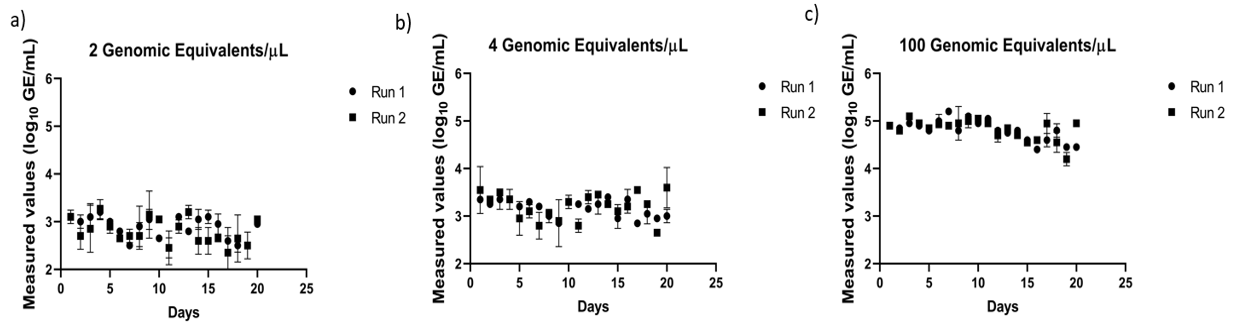

Supplement: Supplementary file 6 — Additional file 6: Figure S3. Daily runs used to estimate within-laboratory precision. Three bacterial concentrations from ATCC 12403 (2, 4 and 100 GE/μL), represented as log10 GE/mL, were evaluated using a single-site design of 20x2x2 according to the CLSI EP05-A3 guidelines. A Values (log10 GE/mL) for the 2 GE/mL concentration obtained from four daily replications over twenty days. B Values (log10 GE/mL) for the 4 GE/mL concentration obtained from four daily replications over twenty days. C Values (log10 GE/mL) for the 100 GE/mL concentration obtained from four daily replications over twenty days. [file 12884_2020_3038_MOESM6_ESM.tif]
